# Lack of Lloviu Virus Disease Development in Ferret Model

**DOI:** 10.3201/eid3012.240818

**Published:** 2024-12

**Authors:** Paige Fletcher, Kyle L. O’Donnell, Joseph F. Rhoderick, Corey W. Henderson, Atsushi Okumura, Trenton Bushmaker, Ayato Takada, Chad S. Clancy, Gábor Kemenesi, Andrea Marzi

**Affiliations:** National Institute of Allergy and Infectious Diseases, Hamilton, Montana, USA (P. Fletcher, K.L. O’Donnell, J.F. Rhoderick, C.W. Henderson, A. Okumura, T. Bushmaker, C.S. Clancy, A. Marzi); Hokkaido University, Sapporo, Japan (A. Takada); University of Pécs, Pécs, Hungary (G. Kemenesi)

## Abstract

The first isolate of the emerging filovirus Lloviu virus (LLOV) was obtained in 2022. No animal disease models have been established. We assessed the pathogenic potential of LLOV in ferrets after intranasal, intramuscular, or aerosol exposure. The lack of disease development shows ferrets are not a disease model for LLOV.

Members of the *Filoviridae* family are negative-sense, nonsegmented, single-stranded RNA viruses with the potential to cause hemorrhagic disease in humans and nonhuman primates. Lloviu virus (LLOV) is an emerging filovirus that is currently the only virus within the *Cuevavirus* genus (*1*). LLOV was first identified in Schreiber’s bats (*Miniopterus schreibersii*) in 2002 in Cueva del Lloviu, Spain (*2*), but no other reports were published until 2016, when it was identified in Schreiber’s bats in Hungary (*3*). LLOV has also been identified in Bosnia-Herzegovina and Italy (4,*5*). The first infectious LLOV isolate was obtained in 2022 (*6*). The human pathogenic potential is currently unknown, although LLOV can infect human cells, which suggests a potential for zoonotic spillover (*6*). No animal disease models have been established to assess LLOV pathogenicity and to better understand this novel filovirus.

Because the ferret model has recently been used to assess wild-type filovirus pathogenesis (*7*), we chose domestic ferrets to investigate the pathogenic potential of LLOV. Mucosal inoculation was of interest because LLOV RNA has been detected in lung samples of infected Schreiber’s bats (*6*). Intranasal (IN), intramuscular (IM), and aerosol inoculations were evaluated to compare different LLOV exposure routes. 

## The Study

We used 18 domestic ferrets (*Mustela putorius furo*) that were 19 weeks old and 0.52–0.97 kg in weight. On 0 days postinfection (dpi), we inoculated the ferrets by either IM (0.4 mL/ferret; 4 female and 2 male), IN (0.3 mL/ferret; 2 female and 4 male), or aerosol (4 female and 2 male) routes of exposure with 1,000 focus-forming units (FFU) of wild-type LLOV (GenBank accession no. OQ630505), as previously described (*8*). We conducted examinations and sample collections on 0, 2, 4, 6, 8, 10, 14, and 21 dpi. At 21 dpi, we humanely euthanized 6 of 18 surviving ferrets (n = 2/exposure group) and repurposed the remaining 12 ferrets to a different study. We conducted analysis by using GraphPad Prism software v. 9.3.1 (GraphPad, https://www.graphpad.com).

We conducted all infectious work with LLOV following standard operating procedures approved by the Rocky Mountain Laboratories (RML) Institutional Biosafety Committee in the maximum containment laboratory at RML, National Institute of Allergy and Infectious Diseases, National Institutes of Health. We conducted animal work in accordance with recommendations described in the Guide for the Care and Use of Laboratory Animals of the National Institutes of Health, the Office of Animal Welfare, and the United States Department of Agriculture. Animal work was approved by the RML Animal Care and Use Committee. Procedures were conducted in animals anesthetized by trained personnel under the supervision of veterinary staff. Ferrets were monitored >1 time/day for clinical signs of disease, including appearance, level of activity, body temperature, and bodyweight determination. Endpoint criteria were used as specified by RML Animal Care and Use Committee approved clinical score parameters.

**Figure 1:**
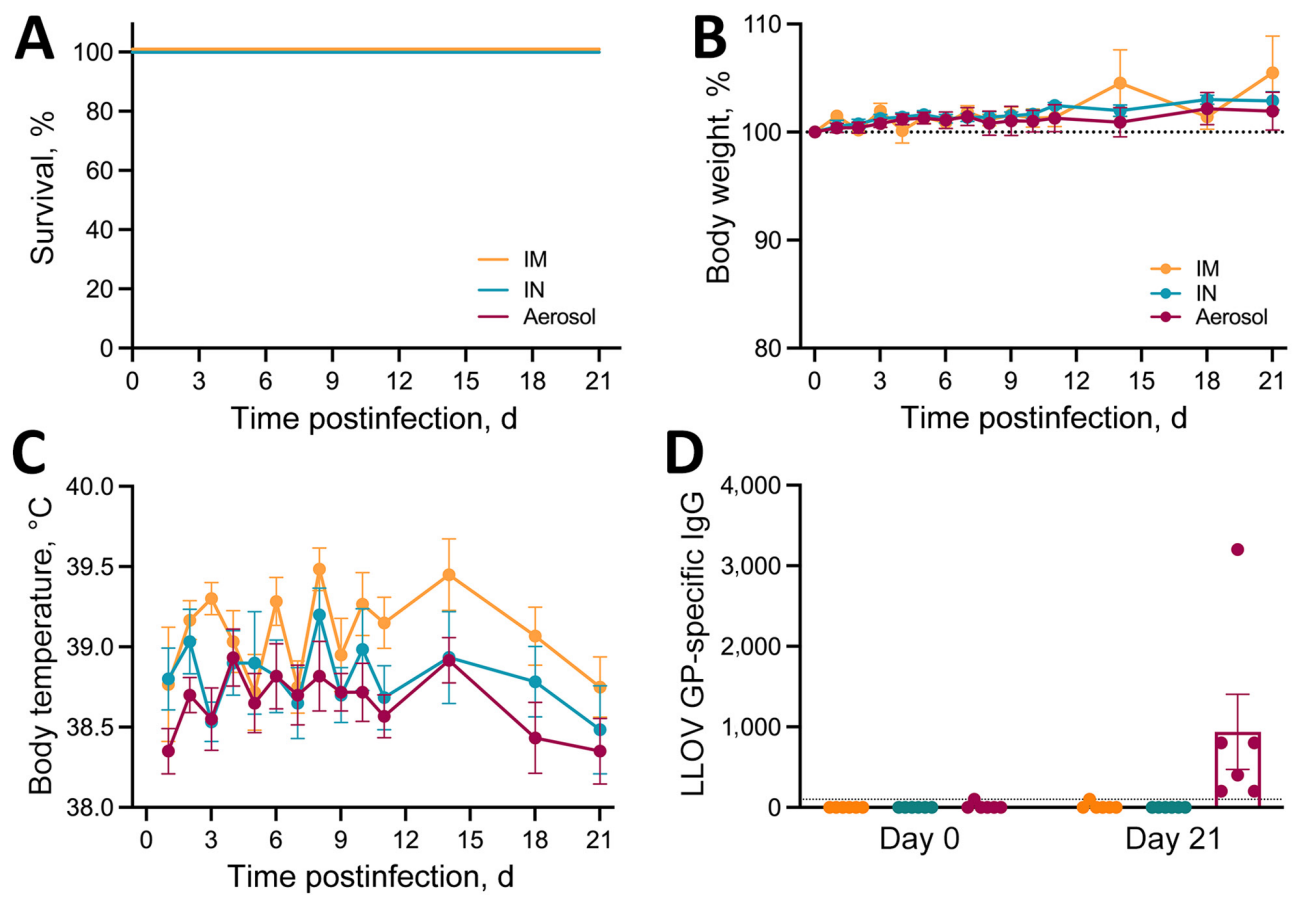
Clinical findings after experimental LLOV exposure in ferrets. Ferrets were inoculated through IM, IN, or aerosol (n = 6/group) routes with 1,000 focus-forming units of LLOV. A) Survival curve. B) Bodyweight percentage; dotted line is normalized to day of infection; error bars indicate standard error of the mean. C) Transponder temperature beginning at 1 day postinfection; error bars indicate standard error of the mean. D) LLOV glycoprotein-specific IgG titers in serum measured by ELISA. Dotted line indicates the limit of detection; error bars indicate standard error of the mean. IM, intramuscular; IN, intranasal; LLOV, Lloviu virus.

We achieved the LLOV target dose for each route of exposure as indicated by LLOV inoculum RNA (Appendix Figure 1, panel A). We determined RNA levels by using a LLOV-specific primer-probe set with extractions and real-time reverse transcription PCR methods as previously described (*8*). All ferrets survived LLOV inoculation (Figure 1, panel A) with no signs of disease regardless of exposure route throughout the study (Figure 1, panel B). Slight fluctuations of temperature levels were detected by transponders that we inserted on 0 dpi (Figure 1, panel C). We detected sporadic LLOV RNA at low levels in the blood, swab, urine, or tissue samples collected throughout the study (Appendix Figure 1, panels B–F). We detected limited titers of LLOV glycoprotein (GP)-specific IgG on 21 dpi in the aerosol-exposed group (*8*) but only negligible amounts in the IM- and IN-exposed ferrets (Figure 1, panel D).

**Figure 2:**
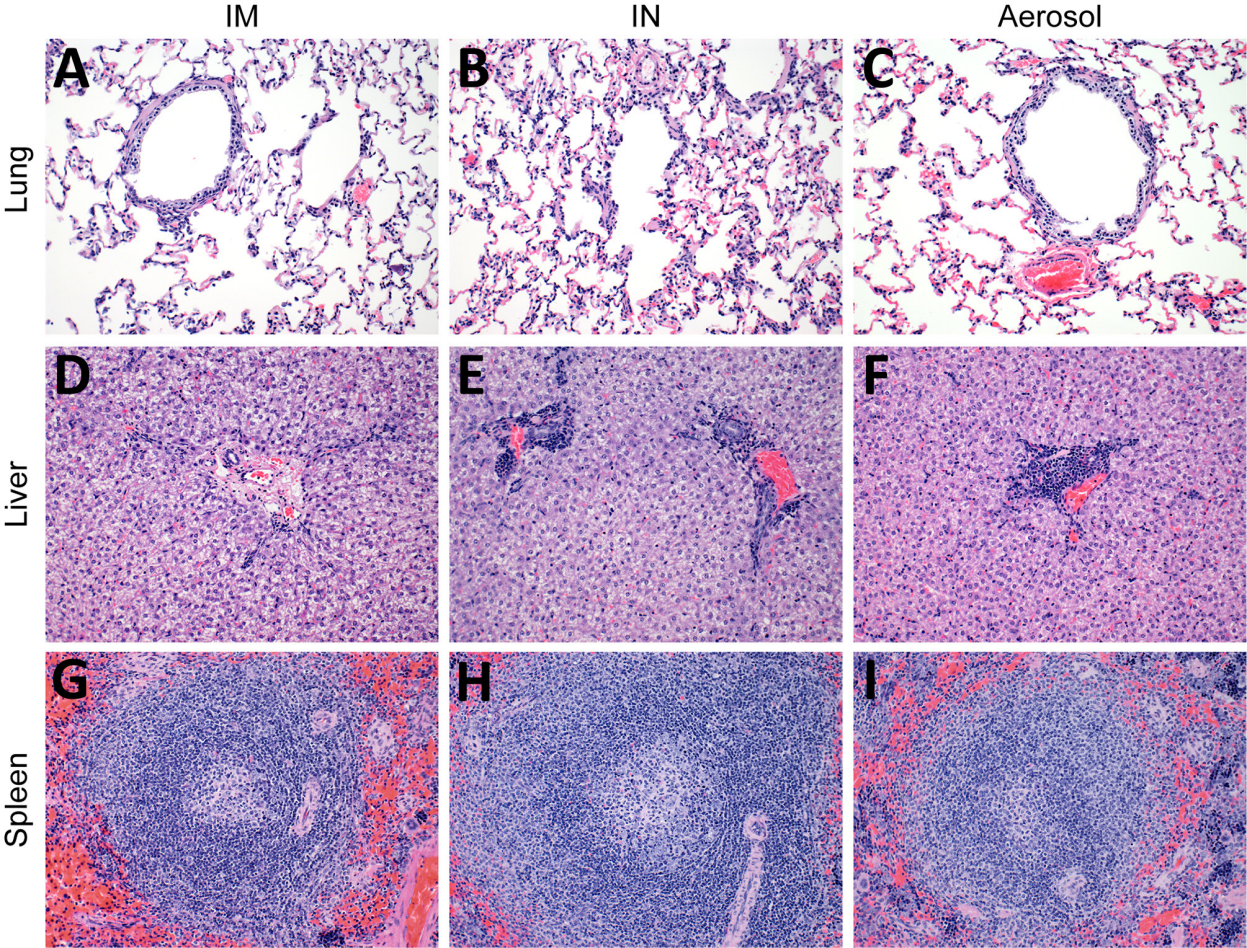
Histopathologic findings after experimental LLOV exposure in ferrets. Ferret tissues showed no abnormalities after LLOV exposure at 21 days postinfection. A–C) Lung tissue; D–F) liver tissue; G–I) spleen tissue. Hematoxylin and eosin staining; original magnification ×​200. Ferrets were inoculated through IM, IN, or aerosol (n = 6/group) routes with 1,000 focus-forming units of LLOV. IM, intramuscular; IN, intranasal; LLOV, Lloviu virus.

We next investigated the hematology and serum biochemistry of LLOV-exposed ferrets, as previously described (*9*). Platelet and lymphocyte (Appendix Figure 2, panels A, B) cell counts remained at normal levels throughout the study regardless of exposure route. Similarly, liver and kidney (Appendix Figure 2, panels C–F) enzyme levels remained normal throughout the study regardless of exposure route, which indicated no clinically relevant organ damage. Those findings were confirmed by histopathologic analysis with hematoxylin and eosin staining, as previously described (*9*). No inflammation or classic filovirus lesions were observed on 21 dpi for any exposure route in the lung, liver, or spleen tissues of the 6 ferrets assessed (Figure 2).

There are no established animal disease models to assess LLOV pathogenicity because it is a novel filovirus. Immunodeficient mice, especially type I interferon receptor knockout (IFNAR^−/−^) mice, have been shown to be susceptible to wildtype filovirus infection (*10*), but a recent study showed that disease did not develop in IFNAR^−/−^ mice after LLOV inoculation regardless of dose and route of exposure (*8*). Domestic ferrets have previously been shown to be susceptible to filovirus infection, and disease developed after IM or IN inoculation with Ebola virus, Bundibugyo virus, Sudan virus, or Reston virus (*7*). Therefore, we assessed LLOV inoculation either by IM, IN, or aerosol routes of exposure in the ferret model.

Our results show the LLOV-exposed ferrets did not develop any signs of disease and survived after exposure, regardless of route, and miniscule amounts of LLOV RNA were detected. Animals maintained normal levels of clinical and hematology parameters throughout the study, and histopathologic assessments indicated no lesions or inflammation. The aerosol group demonstrated the most evidence of LLOV replication and as a result recorded the highest LLOV GP-specific IgG titers. This route of infection was specifically included to deposit the virus directly in the lungs because LLOV was isolated from bat lung samples (*6*). Of note, those results mimic previously published results in the ferret model for IM, IN, or intraperitoneal inoculation of Marburg virus (MARV) and IM or IN inoculation of Ravn virus (*11*,*12*). The differences between filovirus infectivity in ferrets could be related to innate immune evasion in this model. A previous LLOV in vitro study investigated the effect of plasmid-expressed viral proteins (VP) VP24, VP35, and VP40 as innate immune antagonists and found LLOV VP24 and VP35 inhibited interferon responses whereas VP40 did not; although testing was performed in human and bat cells (*Epomops buettikoferi*) and not ferret cells (*13*). That result is of interest because LLOV VP40 is similar to MARV VP40 (*2*); however, MARV VP40 is an interferon antagonist in a species-specific manner (*14*). Further research is needed to assess potential species-specific differences with filovirus infections and their invasion of the innate immune response.

## Conclusions

In this study, all LLOV-exposed ferrets showed no signs of disease, and survival was 100% regardless of exposure route. Our results indicate a limited value for investigation of LLOV pathogenicity and disease development in this model. Our data potentially indicates that LLOV may be less infectious or pathogenic than other previously characterized filoviruses. Those viruses include MARV and Ravn virus, which cause disease in IFNAR^−/−^ mice but not in ferrets, and Ebola virus, Sudan virus, Reston virus, and Bundibugyo virus, which cause lethal disease in ferrets. This result may be because the LLOV isolate used is closely related to the isolate from Hungary. However, LLOV could be less capable of infecting certain animal species, or LLOV infection might occur by specific exposure routes only, in this case by aerosol exposure. Further studies might reveal if LLOV is pathogenic in any animal species other than bats and determine if the virus poses a zoonotic threat. 

Appendix Additional information about lack of Lloviu virus disease development in ferret model.
