# Supplementary material for: Lack of Lloviu Virus Disease Development in Ferret Model
Source: Emerg Infect Dis. 2024 Dec;30(12):2639–42. doi: 10.3201/eid3012.240818 (PMC11616650; doi:10.3201/eid3012.240818)
Supplement: Appendix — Additional information about lack of Lloviu virus disease development in ferret model. [file 24-0818-Techapp-s1.pdf]

Article DOI: https://doi.org/10.3201/eid3012.240818

EID cannot ensure accessibility for supplementary materials supplied by authors. Readers who have difficulty accessing supplementary content should contact the authors for assistance.

Lack of Lloviu Virus Disease Development in Ferret Model

Appendix.

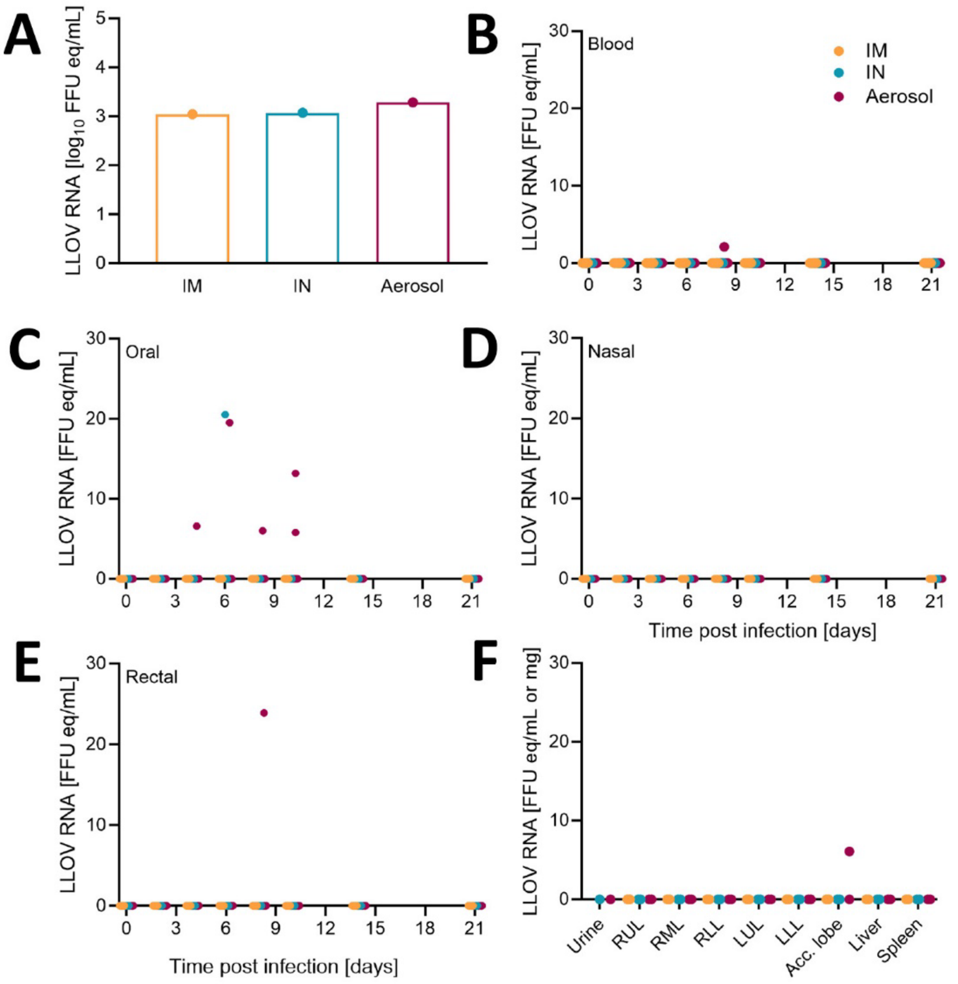

Appendix Figure 1. LLOV load in exposed ferrets. Ferrets were inoculated IM, IN, or aerosol (n = 6/group) with 1,000 FFU of LLOV. (A) LLOV RNA inoculums on 0 dpi in log scale. LLOV RNA in (B) blood, (C) oral swabs, (D) nasal swabs, and (E) rectal swabs throughout the study in

linear scale. (F) LLOV RNA in urine and tissue samples at 21 dpi in linear scale. RUL, right upper lung lobe; RML, right middle lung lobe; RLL, right lower lung lobe; LUL, left upper lung lobe; LLL, left lower lung lobe; Acc. lobe, accessory lung lobe.

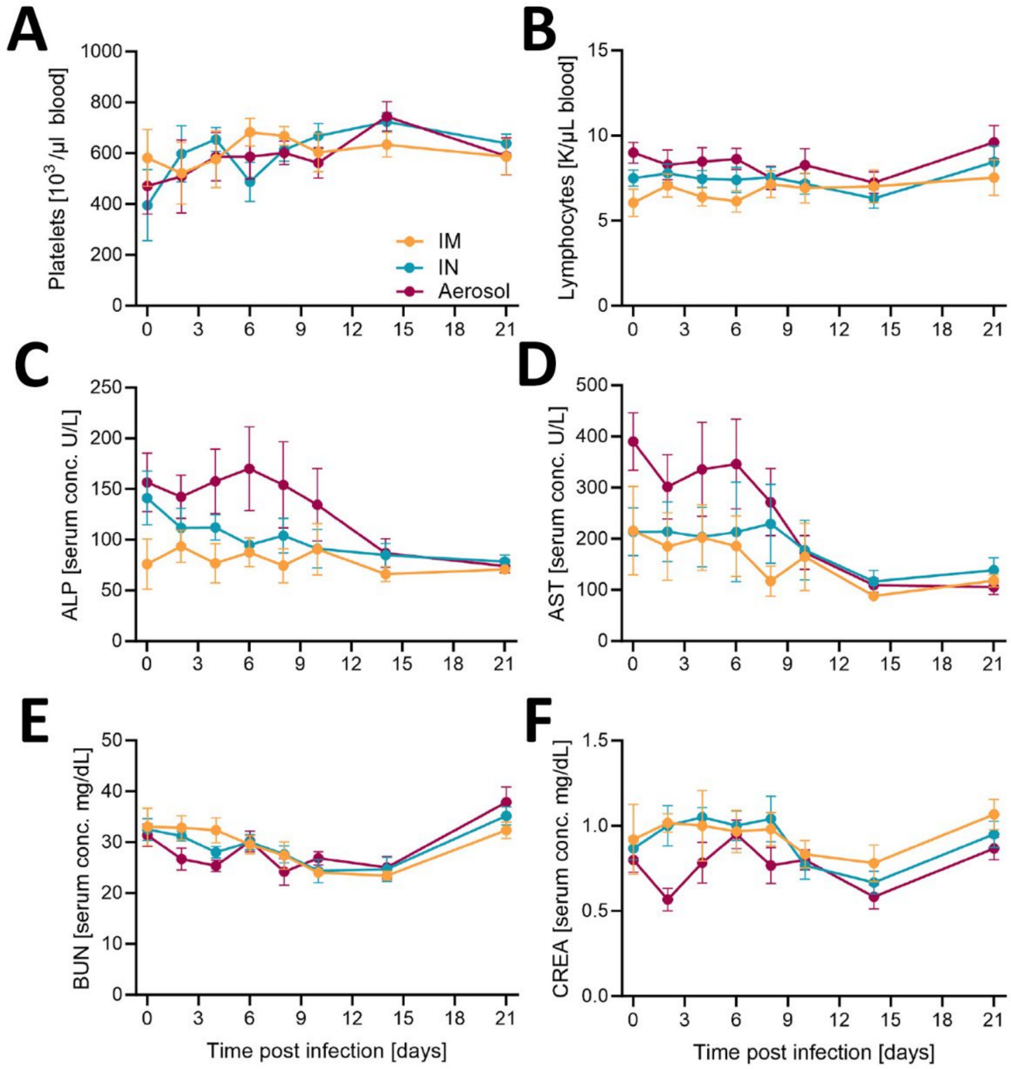

Appendix Figure 2. Select hematology and serum chemistry analysis after LLOV exposure in ferrets. Ferrets were inoculated IM, IN, or aerosol (n = 6/group) with 1,000 FFU of LLOV. (A) Platelet and (B) lymphocyte cell counts in the blood. (C,D) Liver and (E,F) kidney enzyme levels in the serum. LLOV, Lloviu virus; ALP, alkaline phosphatase; AST, aspartate transaminase; BUN, blood urea nitrogen; CREA, creatinine.
